# Gold-incorporated hyaluronic acid nanoparticles enhance ablative radiotherapy efficacy in lung cancer

**DOI:** 10.1016/j.ijpx.2025.100480

**Published:** 2025-12-23

**Authors:** Jenny Ling-Yu Chen, Shu-Jyuan Yang, Li-Cheng Lin, Chun-Kai Pan, Ching-Yi Tsai, Yu-Sen Huang, Ke-Cheng Chen, Ming-Jium Shieh, Yu-Li Lin

**Affiliations:** a Department of Radiology, National Taiwan University College of Medicine, Taipei, Taiwan; b Department of Radiation Oncology, National Taiwan University Cancer Center, Taipei, Taiwan; c Division of Radiation Oncology, Department of Oncology, National Taiwan University Hospital, Taipei, Taiwan; d Institute of Biomedical Engineering, College of Medicine and College of Engineering, National Taiwan University, Taipei, Taiwan; e Department of Medical Research, National Taiwan University Hospital, Taipei, Taiwan; f Department of Medical Imaging, National Taiwan University Hospital, Taipei, Taiwan; g Department of Surgery, National Taiwan University Hospital, Taipei, Taiwan; h Graduate Institute of Toxicology, College of Medicine, National Taiwan University, Taipei, Taiwan

**Keywords:** Lung cancer, Ablative radiotherapy, Gold nanoparticles, Antitumor immunity, Immunogenic cell death

## Abstract

We aimed to investigate the utility of Au-incorporated hyaluronic acid nanoparticles (Au/HA NPs) for improving the therapeutic efficacy of ablative radiotherapy (RT) for tumor control and microenvironment remodeling. HA-functionalized NPs exhibited uniform size, stability, and efficient SN38 encapsulation. Au incorporation increased NP diameter and reduced surface charge while remaining stable. HA and Au/HA NPs were efficiently internalized by lung cancer cells, with free HA pretreatment suppressing internalization. Moreover, Au/HA NP internalization strongly downregulated CD44 expression in lung cancer cells, confirming CD44-mediated internalization. *In vitro*, Au/HA NPs enhanced radiation-induced G2/M phase arrest and γH2AX foci formation with increased DNA double-strand breaks. Au/HA NPs and RT induced immunogenic cell death (ICD) in lung cancer cells, characterized by elevated reactive oxygen species, increased calreticulin surface expression, and extracellular adenosine triphosphate release. Tumor control, survival, immune infiltration, and systemic effects were investigated *in vivo* using A549 xenografts and Lewis lung carcinoma synchronous flank-lung tumor models. Au/HA NPs and ablative RT decreased tumor growth, reduced lung tumor burden in non-irradiated areas, and prolonged survival. This therapeutic combination led to increased infiltration of natural killer (NK), NK T, CD8^+^ T, and dendritic cells and decreased regulatory T cells, suggesting robust immunological activation. Biodistribution studies confirmed CD44-targeted tumor-specific NP accumulation. No substantial toxicity was observed. In conclusion, Au/HA NPs and ablative RT induced ICD *in vivo*. Au/HA NPs enhanced local and systemic immunity *via* radiosensitization and ICD. This NP-assisted approach may improve RT efficacy in lung cancer.

## Highlights

• Au/HA NPs demonstrate radiotherapy-enhanced tumor control and immune response.
• Au incorporation altered the NP size and charge while maintaining stability.
• CD44-mediated uptake is confirmed by downregulated CD44 expression.
• Combination therapy induced ICD and boosted local/systemic immunity.
• Biodistribution showed tumor-specific NP accumulation with no toxicity.

## Graphical Abstract

Schematic overview of the synergistic effects of gold (Au)-incorporated hyaluronic acid (HA) nanoparticle (Au/HA NPs) combined with ablative ionizing radiation in enhancing antitumor immunity. Au/HA NPs are synthesized using a lyophilization–rehydration approach. Upon intravenous administration, these NPs preferentially accumulate in tumors. When combined with ablative ionizing irradiation, Au/HA NPs enhance local cytotoxicity and increase the generation of reactive oxygen species (ROS), thereby promoting immunogenic cell death (ICD). This process triggers the release of damage-associated molecular patterns (DAMPs), including calreticulin (CRT) and ATP, which activate immature dendritic cells (iDCs) and drive their maturation into antigen-presenting mature dendritic cells (mDCs). The resulting presentation of tumor-associated antigens facilitates the recruitment and activation of tumor-infiltrating immune cells (TIIs), including T cells, NK cells, and NKT cells, both at the primary tumor site and in distant tumors, thereby augmenting systemic antitumor immune responses.

Abbreviations: HSA, human serum albumin; HA, hyaluronic acid; NK, natural killer cell; NKT, natural killer T cell

**Figure:**
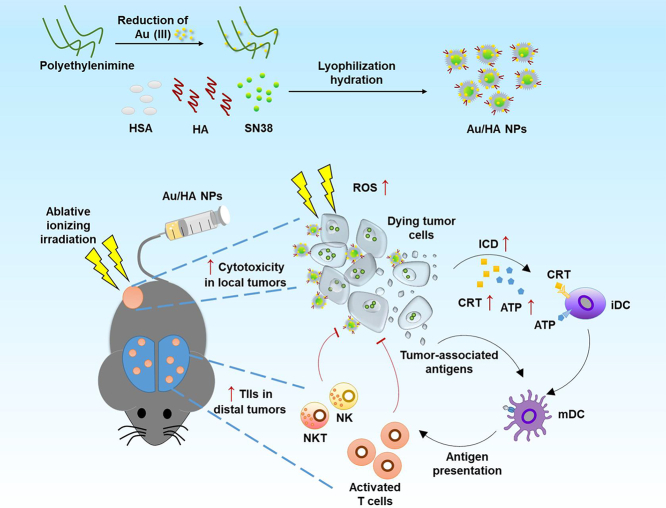

## 1 Introduction

Lung cancer remains the most prevalent malignancy and a leading cause of cancer-related mortality worldwide. Approximately 30–40 % of patients present with metastatic disease at diagnosis, and most are ineligible for surgery (Huang et al., 2019; Islami et al., 2015). However, although surgical options are limited, advances in noninvasive approaches have improved survival. Notably, ablative radiotherapy (RT) provides local control and induces systemic immunity (Huang et al., 2018). This dual benefit is attributed to RT-induced immune activation through the release of proinflammatory mediators, damage-associated molecular patterns (DAMPs), and increased presentation of tumor-associated antigens resulting from immunogenic cell death (ICD) (Chen et al., 2023b; Hernandez et al., 2016).

Irinotecan has potent antitumor activity, and its active metabolite SN38 is 100–1000 times more potent as a topoisomerase I inhibitor. However, the clinical utility of SN38 is limited by its rapid metabolic inactivation and severe gastrointestinal toxicity (Mathijssen et al., 2001). To overcome these challenges, various nanoparticle (NP) formulations have been developed to enhance SN38 stability and bioavailability. Among them, albumin (Alb)-based NPs offer distinct advantages, including high biocompatibility, efficient drug loading capacity, inherent tumor-targeting potential, and facile surface modification (Xiong et al., 2025; Zhang et al., 2013). Hyaluronic acid (HA) is a biocompatible and biodegradable linear polysaccharide that serves as an effective ligand for tumor targeting by binding to cell surface CD44 receptors (Wickens et al., 2017). By mediating receptor-dependent cellular adhesion and endocytosis, HA facilitates selective NP uptake by cancer cells. CD44 is highly expressed in human lung cancers and plays critical roles in epithelial–mesenchymal transition, tumor invasion, and metastatic progression (Chen et al., 2018; Zhang et al., 2020). NP surface modification with HA has therefore emerged as an effective strategy to achieve active, receptor-mediated tumor targeting and enhanced intracellular drug delivery (Kesharwani et al., 2021; Kim and Kumar, 2014).

Metallic-based NPs enhance the efficacy of RT by increasing radiation dose deposition within tumors through their high atomic number (Z), amplifying reactive oxygen species (ROS) generation, and overcoming radioresistance (Chen et al., 2023a; Liu et al., 2018). However, their clinical translation has been limited by potential toxicity, poor colloidal stability, and unintended off-target interactions. Conjugation with polymers overcomes these challenges by improving colloidal stability, prolonging systemic circulation lifetime, and increasing tumor specificity (Beach et al., 2024). Au NPs, with the inherent advantage of biocompatibility and nanoscale dose deposition, amplify radiation effects by releasing secondary electrons and generating ROS. This in turn increases local ionization and exacerbates tumor cell damage (Lo et al., 2023; Shao et al., 2024). Moreover, metallic NPs modulate the tumor microenvironment (TME) by promoting immunogenic cell death (ICD), stimulating both innate and adaptive immune responses (Chen et al., 2023c; Chen et al., 2022; Muradova et al., 2025). The mechanisms include activating the STING pathway; enhancing T cell infiltration and activation; promoting the expression of ICD markers; regulating dendritic cells, macrophages, T cells, and NK cells; increasing tumor-infiltrating immune cell (TIIC) infiltration; and inducing durable systemic antitumor immunity (Li et al., 2024; Shi et al., 2024).

We aimed to evaluate the utility of Au/HA NPs for improving the therapeutic efficacy of RT for TME remodeling in lung cancer. We hypothesized that Au-incorporated HA NPs would enhance the tumor killing, ICD, and local/systemic immune responses. Toward our goal, Au-incorporated HA NPs were developed. By leveraging HA for CD44-targeted delivery and Au NPs for radiosensitization, the formulation was designed to stabilize SN38 and amplify radiation-induced immunogenicity.

## 2 Materials and methods

### 2.1 Materials

SN38 was obtained from ScinoPharm Taiwan, Ltd. (Tainan, Taiwan). Human serum Alb (≥96 %), HA (MW = 500 kDa), branched polyethylenimine (PEI, MW = 25 kDa), chloroauric acid (99.995 %), l-ascorbic acid (99 %), fluorescein 5(6)-isothiocyanate (FITC), and dimethyl sulfoxide (DMSO) were purchased from Sigma–Aldrich (St. Louis, MO, USA).

### 2.2 Au/HA NP preparation

**Fig. 1:**
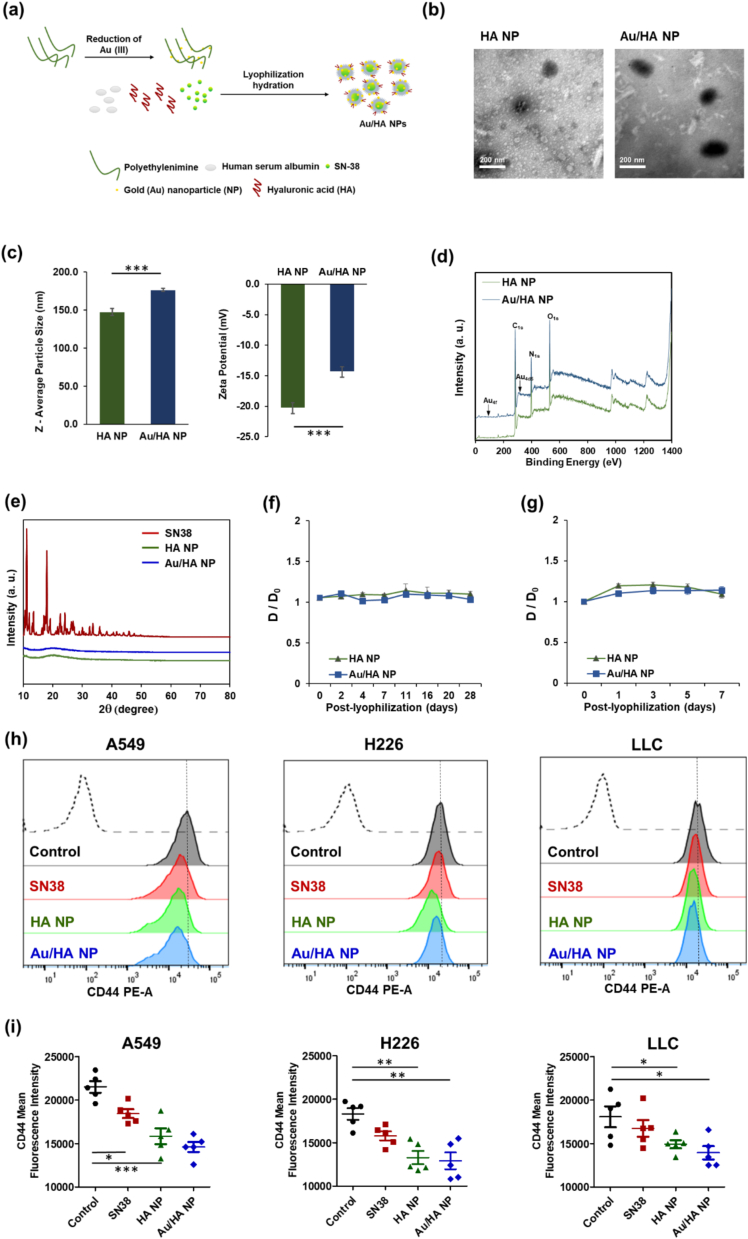
Preparation and characteristics of Au-HA-functionalized SN38 NPs. (a) HA NPs are prepared by complexing SN38 with a human serum albumin (HSA)-based HA/PEI mixture. Simultaneously, Au NP-incorporated HA NPs (Au/HA NPs) are synthesized by integrating PEI-stabilized Au NPs into the HA/albumin complex. HA-functionalized SN38 NPs are prepared using a lyophilization–hydration method. (b) Transmission electron microscopy images of HA NPs and Au/HA NPs. Samples are negatively stained with 2 % uranyl acetate before imaging. Scale bar = 200 nm. (c) The average size and zeta potential of HA NPs and Au/HA NPs are analyzed using dynamic light scattering and aqueous electrophoresis. Each experimental group includes five samples (*n* = 5). Data are expressed as the mean ± standard error of the mean. Statistical analyses are performed using the Mann–Whitney *U* test. (d) Full scan XPS spectrum of HA NPs and Au/HA NPs. (e) X-ray diffraction patterns of SN38, HA NP, and Au/HA NPs. (f) Storage stability of NPs as evaluated according to particle size (D) is determined by dissolving the re-lyophilized HA NPs and Au/HA NPs in 1 mL double-distilled water (ddH_2_O). Each experimental group include five samples (*n* = 5). (g) Colloidal stability of NPs, as measured according to particle size (D), is assessed in Dulbecco's Modified Eagle Medium (Gibco) supplemented with 10 % (*v*/v) fetal bovine serum to evaluate stability under physiologically relevant conditions. Each experimental group includes five samples (n = 5). (h) Representative and (i) quantitative cell surface CD44 expression posttreatment with SN38, HA NPs, and Au/HA NPs in A549, H226, and LLC cells as determined using flow cytometry. Each experimental group includes five samples (n = 5). Data are expressed as the mean ± standard error of the mean. Statistical significance is assessed using the one-way analysis of variance with Tukey's multiple comparisons test. *P < 0.05; **P < 0.01; ***P < 0.001. Abbreviations: HA, hyaluronic acid; LLC, Lewis lung carcinoma; NP, nanoparticle; PEI, polyethyleneimine.

HA NPs were prepared using the lyophilization–hydration method established for hydrophobic drug-encapsulated micelles (Fig. 1a) (Yang et al., 2023). SN38 (0.5 mg) was completely dissolved in 1 mL DMSO, and then an HA/PEI/Alb mixture was added at a weight ratio of 0.16:0.16:20:1 for HA/PEI/Alb/SN38. After lyophilization, the mixture was reconstituted in 1 mL 5 % dextrose solution (Gitose Injection, Nang Kuang Pharmaceutical Co., Tainan, Taiwan) using ultrasonication. Meanwhile, Au/HA NPs were prepared by replacing HA/PEI/Alb with HA/Au-PEI/Alb. Au NPs were synthesized by adding 500 μL PEI solution (2.5 mg/mL) to 15 μL chloroauric acid solution (5 mg/mL) and then mixing vigorously for 10 s. Subsequently, 300 μL l-ascorbic acid solution (10 mg/mL) was introduced, and the mixture was stirred at 100 rpm for 2 h, enabling the reduction of chloroauric acid and the formation of PEI-templated Au NPs. The detailed compositions of the prepared HA NPs and Au/HA NPs are provided in Table S1.

### 2.3 HA NP and Au/HA NP characterization

Powder X-ray diffraction (XRD) patterns of SN38, HA NP, and Au/HA NP were recorded using a D2 PHASER X-ray diffractometer (Bruker Analytical X-Ray Solutions, Madison, WI, USA) equipped with a Cu Kα radiation source (λ = 1.5418 Å). The surface elemental composition of HA NP and Au/HA NPs was characterized by X-ray photoelectron spectroscopy (XPS) using a Hard X-ray Photoelectron Spectroscopy system (ULVAC-PHI Inc., Kanagawa, Japan) equipped with a monochromatic Al Kα source (1.4 keV, 150 W, 10 mA, 15 kV). The morphology of HA NPs and Au/HA NPs was examined using transmission electron microscopy (Hitachi H-7500, Tokyo, Japan). Samples were pre-precipitated, dried onto 200-mesh carbon-coated copper grids, and negatively stained with 2 % uranyl acetate. The particle distribution, average size, polydispersity index, and mean zeta (ζ) potential of the HA NPs and Au/HA NPs were measured using dynamic light scattering and aqueous electrophoresis with a Zetasizer Nano ZS90 (Malvern Instruments Ltd., Malvern, Worcestershire, UK).

The loading efficiency (LE) of SN38 in HA NPs and Au/HA NPs was quantified using high-performance liquid chromatography (HPLC, ACQUITY Arc System, Waters, Milford, MA, USA). Briefly, NP suspensions were filtered through a 0.1-μm Millex GP filter unit (Merck Millipore Ltd., Tullagreen, Ireland) to remove unencapsulated SN38. A 10-μL aliquot of the filtrate was dissolved in DMSO and injected into the HPLC system. Chromatographic separation was performed on a Waters Symmetry® C18 reversed-phase column (XBridge™) using a mobile phase of 25 mM NaH₂PO₄ (pH 3.1) and acetonitrile (1:1, *v*/v) at a flow rate of 1.0 mL/min. SN38 was detected using an ultraviolet (UV) detector at a wavelength of 265 nm (Xuan et al., 2006). Calibration curves were constructed using SN38 standards (0.1–100 μg/mL in DMSO). The LE was calculated as follows:

LE (%) = (total SN38 added – unencapsulated SN38)/total SN38 added × 100.

The storage stability of HA NP and Au/HA NP was assessed by re-lyophilization and storage at 4 °C. The particle size and polydispersity index (PDI) of the reconstituted samples (in 1 mL ddH₂O) were analyzed using a Zetasizer Nano-ZS90 at predetermined time points (days 0, 1, 4, 7, 11, 16, 20, and 28). To evaluate stability under physiologically relevant conditions, the colloidal stability of HA NPs and Au/HA NPs was assessed in Dulbecco's Modified Eagle Medium (DMEM, Gibco) supplemented with 10 % (*v*/v) fetal bovine serum (FBS). Particle size was measured on days 0, 1, 3, 5, and 7 using a Zetasizer Nano-ZS90.

### 2.4 Cell culture

The human lung cancer cell lines A549 (adenocarcinoma) and H226 (squamous cell carcinoma) and the murine Lewis lung carcinoma (LLC) cell line were obtained from the Food Industry Research and Development Institute (Taipei, Taiwan). All cells were cultured in DMEM supplemented with 10 % heat-inactivated FBS and 1 % penicillin-streptomycin. All cell lines were maintained at 37 °C in a humidified incubator with 5 % CO₂ and 95 % air.

### 2.5 Cell viability and cell internalization

Cells were seeded in 96-well plates at 2 × 10^3^ cells/well and treated with different concentrations of SN38, HA NPs, and Au/HA NPs (0, 1, 7.8, 15.6, 31.2, 62.5, 125, 250, 500, 1000, and 4000 ng/mL) for 48 h, with six samples per concentration. Thereafter, 10 μL of the Cell Counting Kit-8 solution (Sigma-Aldrich, Saint Louis, MO, USA) was added, and the cells were further incubated for 2 h. Cell viability was measured according to absorbance at 450 nm. The experiment was repeated thrice. The half-maximal inhibitory concentration (IC_50_) values were calculated using the four parameter sigmoidal dose–response curves generated with GraphPad Prism 5 (GraphPad Software, San Diego, CA, USA). Concentrations of half IC_50_ values were considered as the *in vitro* experimental concentrations for combination treatment with RT in subsequent experiments.

Sterilized glass coverslips were placed in six-well plates seeded with A549, H226, or LLC cells. After overnight attachment, a fresh medium containing FITC-labeled HA NPs or Au/HA NPs (equivalent to 5 μg/mL SN38) was added, and the cells were incubated for 18 h. To evaluate HA–CD44-mediated endocytosis, the cells were pretreated with 0.1 mg/mL HA for 1 h before NP exposure. After incubation, the cells were washed thrice with phosphate-buffered saline (PBS) and fixed in 10 % formalin. Cellular internalization was analyzed using a spectral confocal and multiphoton microscopy system (Leica TCS SP8, Wetzlar, Germany). Intracellular FITC fluorescence was quantified with a Leica Application Suite X software (Leica Microsystems Inc., Deerfield, IL, USA) from six randomly selected regions per sample. For each cell line, the fluorescence intensity of NP-treated groups was normalized to 100 %. In addition, the correlation between CD44 expression and the cellular uptake of HA NPs and Au/HA NPs was further validated using the human colorectal cancer cell line SW620, which had low CD44 expression.

### 2.6 Colony formation

Cells were pretreated with SN38, HA NPs, or Au/HA NPs at half their IC_50_ concentrations; seeded in six-well plates (200–1000 cells/well); and exposed to 0, 2, 4, 6, or 8 Gy γ-radiation at 210 cGy/min using a ^137^Cs Gammacell-40 irradiator (CIS Bio International, Saclay, France). After incubation for 10–14 days, colonies with over 50 cells were fixed with glutaraldehyde, stained with crystal violet (Sigma-Aldrich), and quantified using an ImmunoSpot S6 UV Reader (Cellular Technology Ltd., Shaker Heights, OH, USA).

### 2.7 Flow cytometry for cell surface CD44 expression

A549, H226, and LLC cells were pretreated with SN38, HA NPs, or Au/HA NPs at half their IC_50_ concentrations for 2 h. They were then stained with PE-labeled anti-CD44 antibodies (1:40, BD Biosciences, San Jose, CA, USA) at room temperature (20–22 °C). After washing and centrifugation, samples were analyzed by flow cytometry using the BD FACSLyric System (BD Biosciences), and results were processed with FlowJo v10 (Tree Star, Ashland, OR, USA).

### 2.8 γH2AX analysis

Cells were pretreated for 2 h with SN38, HA NPs, or Au/HA NPs at half their IC_50_ doses and then exposed to 6 Gy RT and collected 8 h later. To assess DNA damage, cells were subjected to Hoechst 33342 (1:2000, Sigma-Aldrich) staining for nuclear visualization. They were also stained with Alexa Fluor 488-conjugated anti-γH2AX (1:500, Sigma–Aldrich) to detect DNA double-strand breaks. Staining was performed in the dark at room temperature. Fluorescence images were captured using the ImageXpress Nano system (Molecular Devices, Sunnyvale, CA, USA) and analyzed with the MetaXpress software (Molecular Devices, Sunnyvale, CA, USA). γH2AX intensity and foci per cell were calculated using the granularity module as total intensity and foci count per cell.

### 2.9 Cell cycle analysis

Cells (10^6^/mL) were irradiated with 6 Gy either alone or after pretreatment with SN38, HA NPs, and Au/HA NPs at half their IC_50_ values. At 24 h post-RT, cells were collected, fixed in 70 % ethanol, and stained with propidium iodide (1:50, Sigma–Aldrich) to assess DNA content. Cell cycle profiles were determined using flow cytometry with the BD FACSLyric System (BD Biosciences). Percentages in subG1, G0/G1, S, and G2/M were quantified with FlowJo v10 (Tree Star).

### 2.10 DCFDA cellular ROS assay

ROS content in lung cancer cells was measured using a DCFDA/H2DCFDA–Cellular ROS Assay Kit (Abcam, Waltham, MA, USA). Cells cultured in a six-well plate (2 × 10^5^/well) or 96-well plate (2 × 10^4^/well) were irradiated with 6 Gy either alone or after SN38, HA NP, and Au/HA NP pretreatment at half their IC_50_ values. The cells were then harvested 24 h post-RT and stained with 5 μM H_2_DCFDA at 37 °C for 30 min to measure cellular ROS levels. After washing with PBS, the fluorescence intensity of ROS was assessed using the BD FACSLyric (BD Biosciences). Flow cytometry analysis was performed using the Clinical Flow Cytometry System (BD Biosciences), with data processed using FlowJo v10 software (Tree Star). ROS images were captured using the ImageXpress Nano Automated Imaging System (Molecular Devices) and analyzed with MetaXpress software (Molecular Devices) for automated quantification.

### 2.11 Calreticulin expression

After pretreatment with SN38, HA NPs, and Au/HA NPs at half their IC_50_ concentrations, cells were irradiated with 6 Gy, harvested 48 h post-RT, and incubated with anti-calreticulin (CRT) antibodies (1:100 dilution, Abcam) for 30 min on ice to determine cell CRT expression. The cells were washed in cold PBS and incubated with an Alexa Fluor 488-conjugated secondary antibody (1:100 dilution, Abcam) for 30 min on ice. Subsequently, they were analyzed *via* fluorescence-activated cell sorting using the BD FACSLyric Clinical Flow Cytometry System (BD Biosciences). Data were analyzed using FlowJo v10 (Tree Star).

### 2.12 Measurement of extracellular adenosine triphosphate (ATP) release

Cells were seeded in six-well plates at a density of 2 × 10^5^ cells per well; pretreated with SN38, HA NPs, or Au/HA NPs at half their IC_50_ concentrations for 2 h; and then irradiated at 6 Gy. At 48 h post-RT, the culture media were collected, transferred to 1.5 mL tubes, and centrifuged to obtain the supernatants. Extracellular ATP levels were measured using the CellTiter-Glo 2.0 Assay kit (Promega, Madison, WI, USA).

### 2.13 Animal experiments

All animal experiments were approved by our Institutional Animal Care and Use Committee (approval number: 20230414). Six-week-old female C57BL/6 and nude mice were obtained from the National Laboratory Animal Center (Taiwan) and maintained under specific pathogen-free conditions in a facility accredited by the Association for Assessment and Accreditation of Laboratory Animal Care. Mice were housed in groups of five per cage, kept on a 12-h light/dark cycle (lights on at 08:00 a.m.), and given free access to sterilized water and standard chow (LabDiet 5001; LabDiet, Gray Summit, MO, USA).

In the A549 flank tumor model, nude mice were subcutaneously injected with 2 × 10^6^ A549 cells in the hind flank. After 14 days, palpable tumors averaging 38 ± 14 mm^3^ formed. Subsequently, the tumor-bearing mice were randomly assigned to one of the following eight treatment groups: control, irinotecan (20 mg/kg on day 0, administered intravenously), HA NP (20 mg/kg SN38 on day 0, administered intravenously), or Au/HA NP (20 mg/kg SN38 on day 0, administered intravenously), with or without ablative RT (12 Gy/day for 2 days on days 0 and 1). The first RT session was initiated within 4 h after intravenous NP administration.

For the synchronous LLC lung and flank tumor model, C57BL/6 mice first received intrapulmonary injections of 2 × 10^3^ LLC cells to establish the lung tumor model. Injections were uniformly administered in the mid-lower region of the right lung in all mice to ensure anatomical consistency. After 3 days, the mice were subcutaneously injected with 2 × 10^5^ LLC cells in the flank. Flank solid tumors (36 ± 13 mm^3^) developed 7 days after inoculation. Tumor-bearing mice were randomized into one of eight groups, as described above in the A549 animal model.

Radiation was delivered using a ^137^Cs Gammacell-40 irradiator (CIS Bio International). Before treatment, mice were anesthetized *via* intramuscular injection of a tiletamine/zolazepam mixture (7.5 mg/kg Zoletil; Virbac, Carros, France) combined with xylazine (0.12 mg/kg Rompun; Bayer, Leverkusen, Germany). To focus the ablative RT on the hind flank tumor while protecting the rest of the body, a custom lead shield with an unshielded window of 1.0 × 0.5 cm was used. Radiation dose delivery and shielding were carefully standardized for all treated mice. The dose rate was set at 210 cGy/min, calibrated using thermoluminescent dosimeters (TLD—100H, Thermo Fisher Scientific, Waltham, MA, USA) and extended dose range films (CareStream, Rochester, NY, USA), following manufacturer instructions and prior protocols (Brodin et al., 2016; Chen et al., 2021). Mice received two fractions of 12 Gy each on consecutive days, totaling 24 Gy, with each session lasting approximately 5.7 min. Tumor size and body weight were measured every 2–3 days. Humane endpoints, based on Institutional Animal Care and Use Committee guidelines, included euthanasia for mice exhibiting ≥20 % weight loss, tumor diameters of ≥20 mm, impaired mobility due to tumor burden, or severe tumor ulceration.

### 2.14 TIIC immunophenotyping

Tumors from the lung and flank were collected 7 days post-RT to isolate TIICs. Tumor tissues were mechanically dissociated into single-cell suspensions using a gentle MACS dissociator and a murine tumor dissociation kit (Miltenyi Biotec, Gladbach, Germany). The resulting cell suspension was further purified through an 80 %/40 % Percoll density gradient (GE Healthcare, Chicago, IL, USA). Spleens were processed separately by gently pressing through a 400-μm stainless-steel mesh to obtain single-cell suspensions. Red blood cells were removed using a hypotonic lysis buffer. The remaining lymphocytes were washed with Hank's balanced salt solution and resuspended in a culture medium containing 10 % FBS. Cells were then stained with fluorescent antibodies targeting CD3, CD8, CD11b, CD11c, CD45, DX5, and F4/80 (BD Biosciences) and analyzed using multiparameter flow cytometry with a BD FACSLyric Clinical Flow Cytometry System (BD Biosciences). Data were processed with FlowJo v10 software (Tree Star).

### 2.15 Biochemical and hematological analyses

Blood samples were obtained from the submandibular vein of mice. Complete and differential blood counts were assessed 7 days post-RT using a hematology analyzer (Exigo H400; Boule Medical, Spånga, Sweden), recording white blood cell count, platelet count, and hemoglobin concentration. Serum creatinine, blood urea nitrogen, alanine aminotransferase, and aspartate aminotransferase levels were measured using a dry chemistry analyzer (DRI-CHEM NX-500; Fujifilm, Tokyo, Japan).

### 2.16 Biodistribution evaluation

To examine the biodistribution of the synthesized NPs, Cy7.0-labeled HA NPs and Au/HA NPs were administered intravenously to LLC tumor-bearing mice. Tissue samples from the tumor, heart, liver, spleen, lungs, and kidneys were collected at 4 and 24 h post-injection and subsequently imaged using an IVIS Imaging System Spectrum Instrument (Xenogen, Caliper Life Sciences Inc. Hopkinton, MA, USA).

### 2.17 CD44 immunohistochemical staining of flank tumors

Mice with A549 flank tumors were euthanized 7 days post-RT, and tumor samples were collected and fixed in 10 % neutral-buffered formalin for histopathological analysis. To evaluate the targeted impact of HA NPs or Au/HA NPs combined with ablative RT, immunohistochemical staining was performed using anti-CD44 antibodies (1:250 dilution, BD Biosciences). CD44 expression was quantitatively analyzed by capturing images from randomly selected fields per condition using a 40× objective lens on a TissueFAXS microscope (TissueGnostics, Wien, Austria). The percentage of the CD44-positive stained area within each observed field was subsequently evaluated.

### 2.18 CRT immunohistochemical staining of flank tumors

Mice with LLC tumors implanted in the flank were euthanized 7 days post-RT, and tumor samples were harvested and fixed in 10 % neutral-buffered formalin for histopathological evaluation. To investigate ICD responses, specifically CRT translocation to the surface of dying tumor cells following combination treatment with Au/HA NPs and ablative RT, tumor sections were stained immunohistochemically using anti-CRT antibodies (1:500 dilution, Abcam). CRT expression was quantitatively analyzed by capturing images from randomly selected fields per condition using a 40× objective lens on a TissueFAXS microscope (TissueGnostics). The percentage of the CRT-positive stained area within each observed field was subsequently evaluated.

### 2.19 Statistical analysis

Data are presented as the mean ± standard error of the mean (SEM). The exact sample size (n) for each experimental group is explicitly stated in the corresponding figure legends. Comparisons between two groups were analyzed using the non-parametric Mann–Whitney *U* test. For comparisons among three or more groups, statistical significance was determined using one-way analysis of variance (ANOVA) followed by Tukey's post-hoc test to correct for multiple comparisons. Survival analysis was performed using the Kaplan–Meier method, and differences between curves were assessed using the log-rank (Mantel–Cox) test. All statistical analyses were performed using GraphPad Prism 5.01 software (San Diego, CA, USA). A *P*-value of ≤0.05 was considered significant.

## 3 Results

### 3.1 Characteristics of Au-incorporated HA nanoparticles

The gold-incorporated HA nanoparticles were prepared using the lyophilization–hydration method. HA NPs and Au/HA NPs were spherical, with an average diameter of 147.6 ± 4.5 nm and 176.6 ± 1.9 nm (*P* < 0.001), respectively, and a ζ potential of −20.3 ± 0.9 mV and − 14.4 ± 0.8 mV, respectively (*P* < 0.001) (Fig. 1b, c). These results demonstrate that the incorporation of Au resulted in larger NPs with a less negative surface charge. The PDI of HA NPs and Au/HA NPs was 0.200 ± 0.003 and 0.185 ± 0.012, respectively, indicating size uniformity. Furthermore, the particle size distributions of HA NPs and Au/HA NPs across three independent batches showed no significant variation (Fig. S1), demonstrating the robustness, stability, and reproducibility of the preparation method. SN38 is hydrophobic and crystalline. The LE (%) of SN38 in HA NPs and Au/HA NPs was 93.8 ± 5.09 and 95.0 ± 4.13, respectively. XPS analysis confirmed the presence of C, O, N, and Au elements in the Au/HA NPs.

However, the low Au NP incorporation resulted in weak Au signals at 84 eV (Au _4f_) and 335 eV (Au _4d5_), making it difficult to clearly differentiate them from HA NPs (Fig. 1d). XRD pattern can be used to assess its physical state in gold-incorporated HA nanoparticles. The sharp diffraction peaks observed in pure SN38 (Fig. 1e), indicative of its strong crystallinity, disappeared in the XRD patterns of HA NPs and Au/HA NPs. This suggested successful encapsulation within the NPs, likely in a reduced crystalline or amorphous form. With respect to storage stability, re-lyophilized HA NPs and Au/HA NPs showed no significant variations in particle size or PDI value over 1 month (Fig. 1f and Fig. S2), confirming their good stability during preservation at 4 °C. In addition, HA NPs and Au/HA NPs remained stable in DMEM supplemented with 10 % FBS for up to 7 days (Fig. 1g and Fig. S2). This indicated minimal nonspecific protein adsorption and the absence of significant aggregation under physiologically relevant conditions.

### 3.2 CD44 expression and cellular toxicities

To investigate CD44 expression, three lung cancer cell lines, A549 human adenocarcinoma, H226 human squamous cell carcinoma, and LLC murine lung carcinoma cells, were stained with CD44 antibody and analyzed using flow cytometry. The result showed varying levels of CD44 expression varied among the three lung cancer cell lines, with the expression being the highest in A549 cells, followed by that in H226 cells. LLC cells exhibited moderate expression (Fig. 1h, i). As shown in Fig. S3, both HA NPs and Au/HA NPs were internalized by A549, H226, and LLC cells. This internalization was markedly suppressed by pretreatment with 0.1 mg/mL free HA, indicating that cellular internalization occurred predominantly *via* CD44-mediated endocytosis. SW620 cells, which had low CD44 expression (Yang et al., 2023), showed no appreciable change in green fluorescence intensity following 0.1 mg/mL HA pretreatment in either the HA NP– or Au/HA NP–treated group. This indicated that the limited CD44 expression resulted in minimal CD44-mediated NP uptake. Furthermore, the degree of inhibition by free HA correlated with CD44 expression across all cell lines, being highest in A549 cells (∼30 %), moderate in H226 cells (∼25 %), and lowest in LLC cells (∼18 %). These findings were consistent with those of the flow cytometry analysis (Fig. 1h, i). Free SN38 reduced CD44 protein expression, and Au/HA NP internalization significantly downregulated CD44 expression in lung cancer cell lines, confirming active CD44 engagement and NP endocytosis (Fig. 1h, i).

**Fig. 2:**
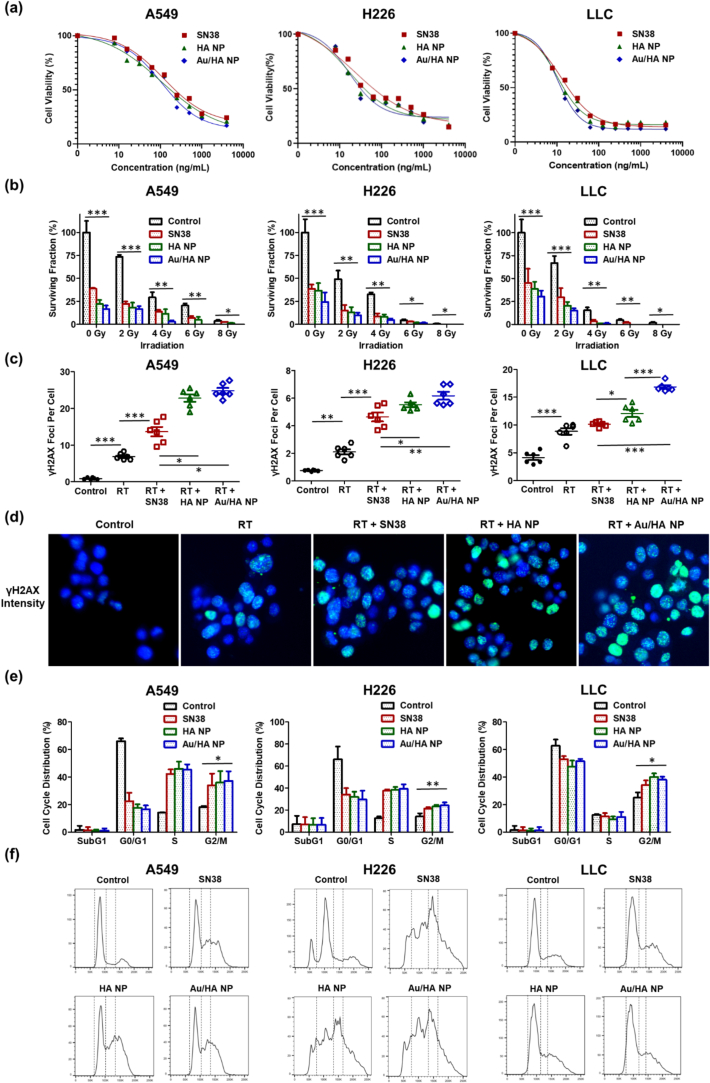
*In vitro* cytotoxicity and radiosensitization effects of Au-hyaluronic acid (HA)-functionalized SN38 NPs in A549 (human adenocarcinoma), H226 (human squamous cell carcinoma), and Lewis lung carcinoma (murine lung carcinoma) lung cancer cell lines. (a) *In vitro* cytotoxicity effect of SN38, HA NPs, and Au/HA NPs in A549, H226, and LLC cells. Cells are treated with different concentrations of SN38, HA NPs, and Au/HA NPs. Cell viability is determined using a Cell Counting Kit-8 (Sigma–Aldrich, Saint Louis, MO, USA) assay. Each experimental group includes six samples per concentration (*n* = 6). (b) Response of lung cancer cells to radiation (0, 2, 4, 6, or 8 Gy) in the presence of SN38, HA NPs, and Au/HA NPs, as determined *via* colony formation assay. Each experimental group includes five samples (n = 5). (c) Quantification of γH2AX foci per cell, as a marker of DNA double-strand breaks after exposure to ionizing radiation, in A549, H226, and LLC cells treated with SN38, HA NPs, or Au/HA NPs and ablative radiotherapy (RT, 6 Gy). Each experimental group includes six samples (n = 6). (d) Representative immunofluorescence merged images of γ-H2AX foci shown in green (AlexaFluor488) and nuclei in blue (Hoechst 33342) in LLC cells treated with SN38, HA NPs, or Au/HA NPs and 6-Gy RT. (e) Analysis of cell cycle distribution. Each experimental group comprised five samples (n = 5). (f) Representative cell cycle distribution of A549, H226, and LLC cells treated with SN38, HA NPs, or Au/HA NPs and 6 Gy RT, as analyzed *via* flow cytometry. Data are expressed as the mean ± standard error of the mean. Statistical significance is assessed using the one-way analysis of variance with Tukey's multiple comparisons test. *P < 0.05; **P < 0.01; ***P < 0.001. Abbreviations: HA, hyaluronic acid; LLC, Lewis lung carcinoma; NP, nanoparticles; RT, radiotherapy. (For interpretation of the references to colour in this figure legend, the reader is referred to the web version of this article.)

The *in vitro* cytotoxicities of HA NPs and Au/HA NPs were compared with those of free SN38. The cell viability assays showed that the average IC_50_ values from three independent experiments of free SN38, HA NPs, and Au/HA NPs were 119.7, 106.1, and 108.4 ng/mL for A549 cells; 20.4, 16.6, and 15.4 ng/mL for H226 cells; and 12.8, 10.4, and 10.4 ng/mL for LLC cells, respectively (Fig. 2a). The calculated IC_50_ values with 95 % confidence intervals are shown in Table S2. The lower IC_50_ values of HA-targeted NPs than that of free SN38 may suggest improved cellular delivery of SN38. The *in vitro* responses of tumor cells to RT in the presence of free SN38, HA NPs, and Au/HA NPs were demonstrated. The surviving fraction was significantly lower with RT combined with free SN38, HA NPs, or Au/HA NPs than with RT alone. This demonstrated that both SN38 and its NP forms effectively enhanced the antitumor effects of RT in a radiation dose-dependent manner. Additionally, adding Au to HA NPs further increased their antitumor effects (Fig. 2b).

### 3.3 Au/HA NP-induced radiosensitization was mediated by increased DNA double-strand breaks and G2/M phase arrest

In A549, H226, and LLC cells, combining RT with free SN38, HA NPs, or Au/HA NPs led to a significant increase in γH2AX foci per cell (Fig. 2c). In LLC cells, γH2AX foci counts post-RT were further increased in the Au/HA NP group (Fig. 2d). Flow cytometry cell cycle analysis showed that free SN38, HA NPs, and Au/HA NPs all altered cell cycles, leading to cell cycle arrest in the G2/M phase (Fig. 2e, f). As cancer cells are most radiosensitive in the G2/M phase, chemotherapeutics driving cells from the G0/G1 phase to the G2/M phase may enhance the antitumor effects of RT.

### 3.4 Au/HA NPs enhanced radiation-induced ICD

**Fig. 3:**
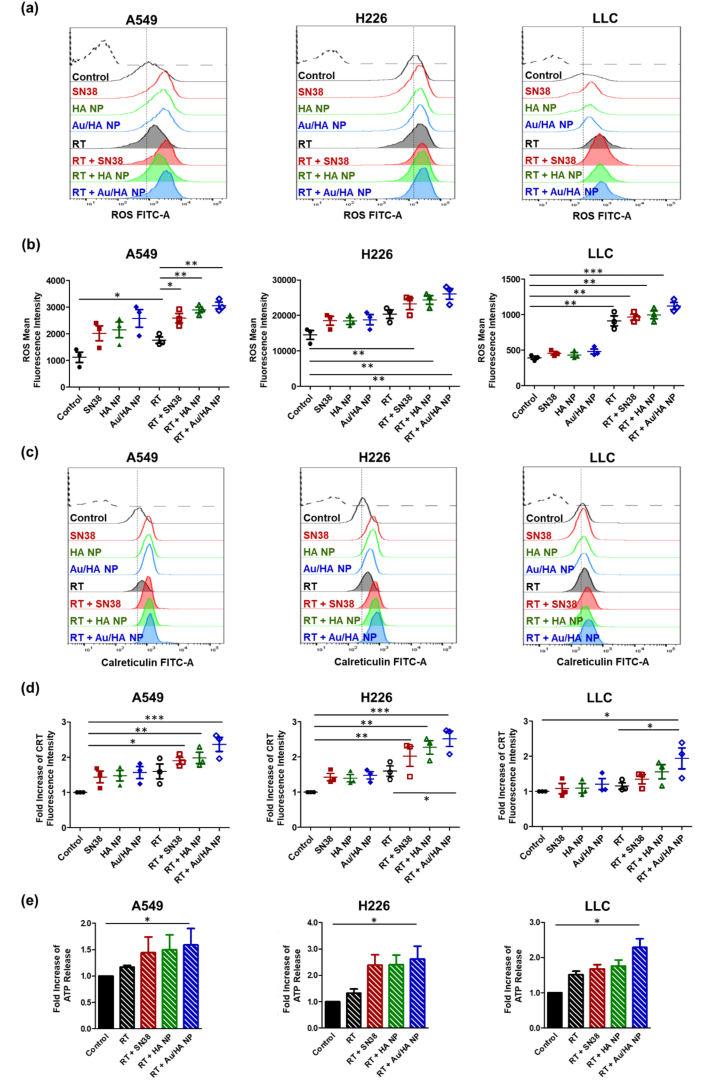
Radiation-induced ICD through ROS generation, CRT expression, and extracellular ATP release. ROS generation induces nuclear damage and triggers ICD. Intracellular ROS are detected by DCFH-DA staining. (a) Representative ROS generation and (b) quantitative intracellular ROS generation after treatment with SN38, HA-functionalized SN38 NPs, Au-HA NPs, or RT (6 Gy) in H226 and LLC cells, as determined using flow cytometry. Each experimental group includes three samples (*n* = 3). (c) Representative CRT expression. (d) Quantification of CRT expression in lung cancer cells after RT combined with SN38, HA NPs, or Au/HA NPs, as determined using flow cytometry. Each experimental group includes three samples (n = 3). (e) Quantification of extracellular ATP release of lung cancer cells after RT treatment combined with SN38, HA NPs, or Au/HA NPs. Each experimental group includes three samples (n = 3). Data are expressed as the mean ± standard error of the mean. Statistical significance is assessed using the one-way analysis of variance with Tukey's multiple comparisons test. *P < 0.05; **P < 0.01; ***P < 0.001. Abbreviations: CRT, calreticulin; HA, hyaluronic acid; ICD, immunogenic cell death; LLC, Lewis lung carcinoma; NPs, nanoparticles; ROS, reactive oxygen species; RT, radiotherapy.

Radiation-induced ICD involves ROS generation, CRT expression, and extracellular ATP release. Flow cytometry was used to assess the impact of free SN38, HA NPs, and Au/HA NPs on ROS production in irradiated lung cancer cells. Both the flow cytometry (Fig. 3a, b) and fluorescence intensity (Fig. S4) data demonstrated substantial ROS generation in lung cancer cells following the combination of Au/HA NPs and RT. This suggested that this treatment strategy induced a strong oxidative stress response in lung cancer cells. ICD responses are characterized by the translocation of CRT to the surface of dying tumor cells, where it functions as an “eat me” signal for antigen-presenting cells (APCs). CRT expression was significantly increased with RT alone, but it was further increased when RT was combined with free SN38. However, Au/HA NPs induced the most pronounced CRT expression among lung cancer cells (Fig. 3c, d). Particularly, flow cytometric analysis of CRT expression on the lung cancer cell surface demonstrated that the combination of RT and Au/HA NPs potently induced ICD responses. Extracellular ATP release increased following RT alone (Fig. 3e) and was further increased with the addition of free SN38. Importantly, extracellular ATP release in lung cancer cells peaked with Au/HA NP treatment. This enhanced ROS generation, CRT expression, and extracellular ATP release likely reinforced ICD-mediated pathways, ultimately promoting robust systemic antitumor immunity.

### 3.5 Au/HA NPs enhanced antitumor efficacy after ablative RT and prolonged survival

**Fig. 4:**
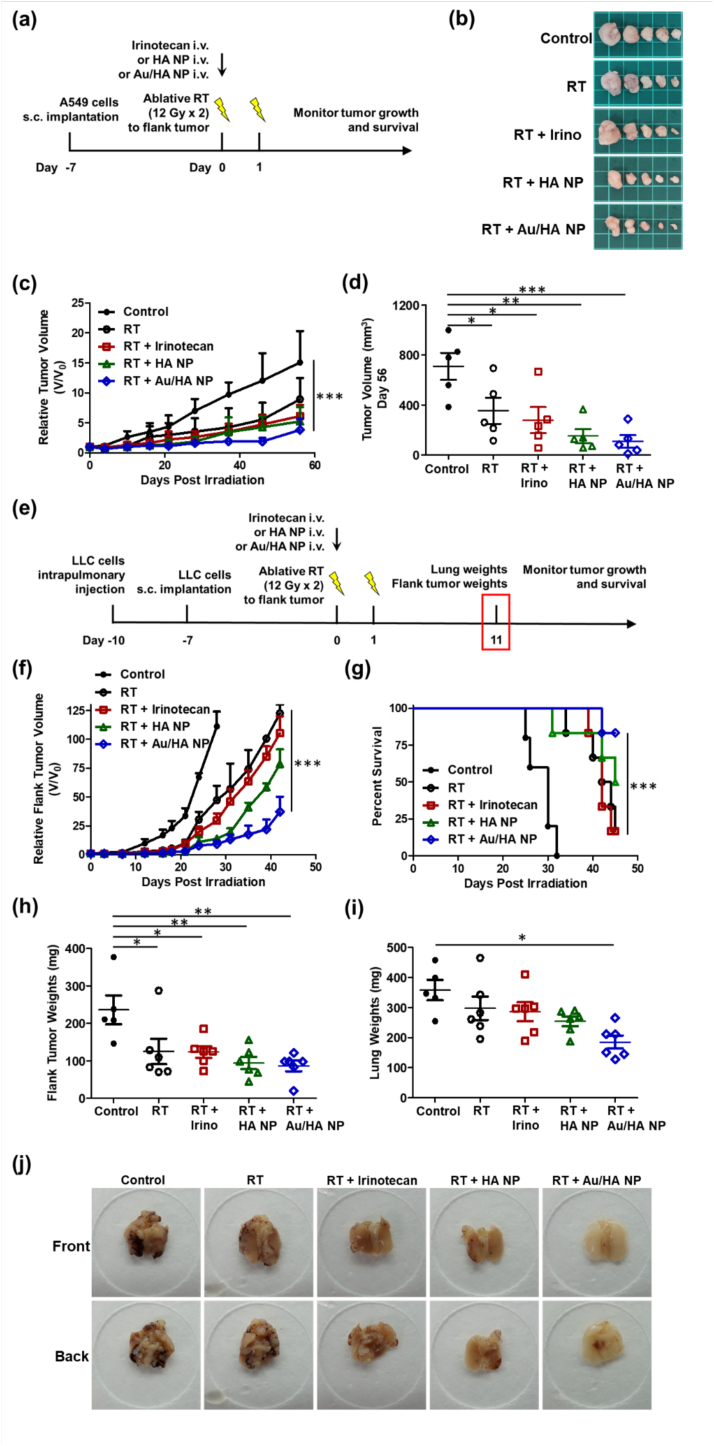
*In vivo* antitumor effect of Au/HA-functionalized SN38 NPs combined with RT. (a) Nude mice are subcutaneously implanted with human adenocarcinoma (A549) cells (2 × 10^6^) on the flanks. Flank solid tumors (38 ± 14 mm^3^) develop 14 days after inoculation. Mice are then randomized to treatment: irinotecan (20 mg/kg on day 0, administered intravenously), HA SN38 NPs (20 mg SN38/kg on day 0, administered intravenously), or Au-modified HA SN38 NPs (20 mg/kg SN38 on day 0, administered intravenously), with or without ablative RT (12 Gy/day for 2 days on days 0 and 1) to the flank tumor. Each experimental group involves five mice (n = 5). (b) Tumor images, (c) relative tumor growth curves, and (d) tumor volumes on day 56 post-RT of the subcutaneous A549 xenograft flank tumor model. (e) Synchronous LLC lung and flank tumor model. C57BL/6 mice undergo intrapulmonary injection and flank subcutaneous implantation with murine LLC cells. C57BL/6 mice first receive an intrapulmonary injection of 2 × 10^3^ LLC cells for tumor development. After 3 days, mice are subcutaneously injected with 2 × 10^5^ LLC cells in the flank. Flank solid tumors (36 ± 13 mm^3^) develop 7 days after inoculation. Tumor-bearing mice are randomized into eight groups, as described above. The control group consisted of five mice (n = 5), whereas each treatment group consisted of six mice (n = 6). (f) Relative flank tumor growth curves and (g) Kaplan–Meier curves of overall survival for the synchronous LLC lung and flank tumor model mice. Mice are euthanized 3 weeks after intrapulmonary injection of LLC cells to assess both lung and flank tumor burdens, (h) flank tumor weights, and (i) lung weights as a proxy of intrapulmonary tumor burden and to obtain (j) lung images from the front and back. Data are expressed as the mean ± standard error of the mean. Statistical analyses are performed using the one-way analysis of variance with Tukey's multiple comparisons test. *P < 0.05; **P < 0.01; ***P < 0.001. Abbreviations: HA, hyaluronic acid; i.v., intravenous; LLC, Lewis lung carcinoma; NP, nanoparticle; RT, radiotherapy; s.c., subcutaneous.

To investigate whether HA NPs and Au/HA NPs can improve the local antitumor effects of ablative RT, the method for creating the subcutaneous A549 xenograft flank tumor model is shown in Fig. 4a. The A549 flank tumor volume at the start of treatment was 38 ± 14 mm^3^. Treatments included irinotecan (20 mg/kg on day 0, administered intravenously), HA NP (20 mg/kg SN38 on day 0, administered intravenously), and Au/HA NP (20 mg/kg SN38 on day 0, administered intravenously), with or without ablative RT (12 Gy/day for 2 days on days 0 and 1). Ablative RT effectively delayed flank tumor growth, and the combination of RT with irinotecan, HA NPs, or Au/HA NPs further enhanced tumor control, as evidenced by reduced tumor volume on day 56 (Fig. 4b–d).

To investigate the immunotherapeutic efficacy and abscopal effects of ICD induced by combining ablative RT with HA NPs and Au/HA NPs, the method for developing the syngeneic synchronous LLC lung and flank tumor model is shown in Fig. 4e. The LLC flank solid tumor volume at the start of treatment was 36 ± 13 mm^3^, and the treatment were the same as described above: irinotecan (20 mg/kg on day 0, administered intravenously), HA NP (20 mg/kg SN38 on day 0, administered intravenously), and Au/HA NP (20 mg/kg SN38 on day 0, administered intravenously), with or without ablative RT (12 Gy/day for 2 days on days 0 and 1). Ablative RT alone or in combination with irinotecan, HA NPs, or Au/HA NPs suppressed flank tumor growth by day 24 (Fig. 4f, g). Ablative RT alone or with irinotecan failed to sustain tumor suppression, with the tumor regrowing by day 42. In contrast, ablative RT combined with Au/HA NPs exhibited prolonged and effective tumor control (Fig. 4g).

To evaluate the systemic immunogenic antitumor response following ablative RT, we assessed non-irradiated lung tumors, using lung weight as a proxy for intrapulmonary tumor burden. Mice were euthanized 3 weeks after intrapulmonary LLC cell injection to assess both lung and flank tumor burdens. While the growth of the flank tumor was effectively controlled with ablative RT alone or combined with irinotecan (Fig. 4h), there was limited efficacy for non-irradiated lung tumors (Fig. 4i). Notably, combining ablative RT with Au/HA NPs significantly enhanced systemic antitumor immunity and reduced intrapulmonary tumor burden. This was evidenced by the lowest lung weights in the combined ablative RT and Au/HA NP group, indicating a pronounced abscopal antitumor effect (Fig. 4i). These effects on flank tumors and lung tumors were reflected in overall survival, with mice in the combined ablative RT and Au/HA NP group demonstrating the longest survival.

### 3.6 Ablative RT combined with Au/HA NPs increased local immune cell infiltration in the flank TME

**Fig. 5:**
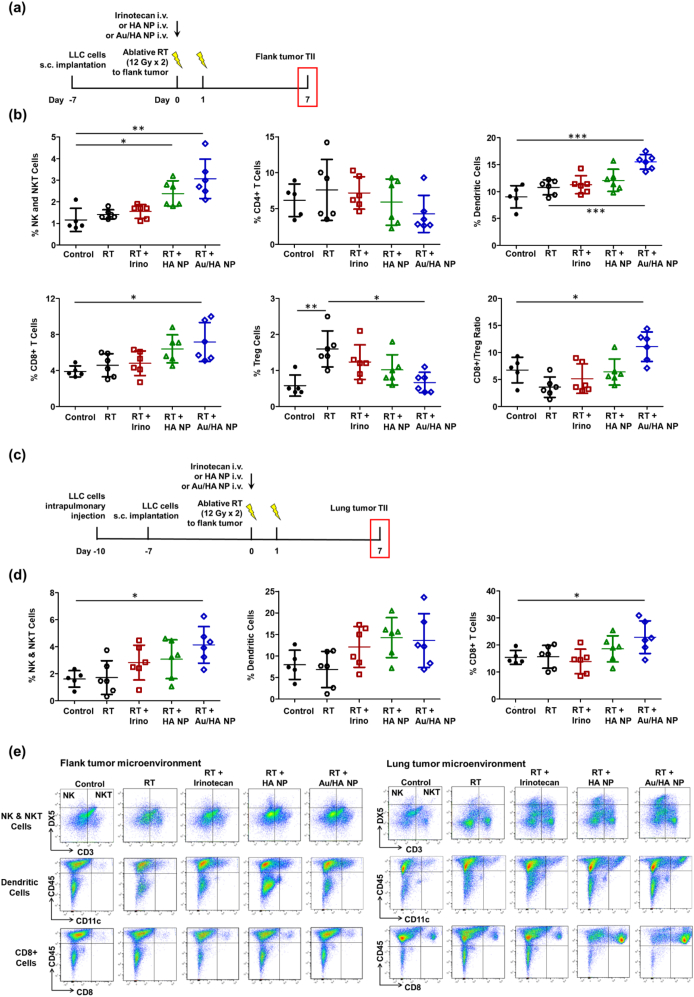
Quantitative flow cytometric analysis of subpopulations of tumor-infiltrating immune cell in the flank and lung tumor microenvironment. (a) C57BL/6 mice undergo subcutaneous implantation of murine LLC cells in the flanks and are randomized to treatment: irinotecan (20 mg/kg on day 0, administered intravenously), hyaluronic acid SN38 nanoparticles (HA NPs, 20 mg SN38/kg on day 0, administered intravenously), or Au-modified HA SN38 NPs (Au/HA NPs, 20 mg SN38/kg on day 0, administered intravenously), with or without ablative radiotherapy (12 Gy/day for 2 days on days 0 and 1) to the flank tumor. TIICs are isolated from the flank tumor microenvironment to obtain cell suspensions for surface staining. (b) Quantitative data of the percentage of tumor-infiltrating immune cells in the flank tumor environment, CD3^−^CD49b^+^ NK cells and CD3^+^CD49b^+^ NK T cells, CD4^+^ T cells, CD11c^+^ dendritic cells, CD8^+^ T cells, and CD4^+^ FoxP3^+^ regulatory T cells and the ratio of CD8^+^ T cells and regulatory T cells are shown. (c) C57BL/6 mice receive intrapulmonary injections of murine LLC cells, undergo subcutaneous implantation with murine LLC cells in the flanks, and are randomized to treatment as described above. Tumor-infiltrating immune cells are isolated from the non-irradiated lung tumor microenvironment to obtain cell suspensions for surface staining. (d) Quantitative data of the percentage of tumor-infiltrating immune cells in the non-irradiated lung tumor microenvironment, CD3^−^CD49b^+^ NK cells and CD3^+^CD49b^+^ NK T cells, CD11c^+^ dendritic cells, and CD8^+^ T cells are shown. (e) Representative flow cytometric analysis, CD3^−^CD49b^+^ NK cells and CD3^+^CD49b^+^ NK T cells, CD11c^+^ dendritic cells, and CD8^+^ T cells in the flank tumor microenvironment (left panel) and the non-irradiated lung tumor microenvironment (right panel) are shown. The control group consisted of five mice (n = 5), whereas each treatment group consisted of six mice (n = 6). Data are expressed as the mean ± standard error of the mean. Statistical analyses are performed using the one-way analysis of variance with Tukey's multiple comparisons test. *P < 0.05; **P < 0.01; ***P < 0.001. Abbreviations: HA, hyaluronic acid; i.v., intravenous; LLC, Lewis lung carcinoma; NK, natural killer cell; NKT, natural killer T cell; NP, nanoparticle; RT, radiotherapy; s.c., subcutaneous.

The infiltration of immune effector cells into tumor sites plays a vital role in enhancing the TME following radiotherapy. Natural killer (NK) cells, as key players in the innate immune response, act as the initial defenders against cancer cell invasion. Meanwhile, T cells constitute the central elements of the adaptive immune system, orchestrating a targeted and sustained antitumor response. On day 7 posttreatment, the percentages of DX5^+^/CD3^−^ NK cells, DX5^+^/CD3^+^ NKT cells, and CD8^+^ T cells remained unchanged in the ablative RT alone group with reference to those in the control group (Fig. 5a, b, e). This indicated that RT alone had minimal effects on the intra-tumoral recruitment of cytotoxic effector cells. The combination of ablative RT and HA NPs or Au/HA NPs increased the number of cytotoxic TIICs, as evidenced by an increased proportion of NK and CD8^+^ T cells. Importantly, the greatest increase was observed in the RT combined with Au/HA NP group. This indicated that the addition of Au further enhanced the immunogenicity of the irradiated TME. With respect to the T cell subpopulations, while ablative RT alone increased the accumulation of regulatory T cells in the TME, the combination of ablative RT and Au/HA NP was associated with a decreased number of regulatory T cells and significantly increased CD8^+^/FoxP3^+^ ratio (Fig. S5).

APCs are essential for triggering adaptive immunity by processing tumor-derived proteins and presenting peptide fragments to TIIs, thereby activating their For cytotoxicity, ablative RT alone induced only a modest increase in the infiltration of dendritic cells (CD11c^+^) within the TME (Fig. 5a, b, e). In contrast, ablative RT combined with HA NPs or Au/HA NPs was associated with a significantly elevated proportion of these antigen-presenting dendritic cells. This enhanced recruitment of APCs, together with the greater accumulation of cytotoxic TIICs observed following the combined treatment, indicated that the ICD induced by this regimen may promote a stronger and more effective adaptive antitumor immune response.

### 3.7 Immunogenicity potential of ablative RT combined with Au/HA NPs in distal lung tumors

To investigate TIIs in distant non-irradiated lung tumors after treatment with combined HA NPs or Au/HA NPs and ablative RT, the method for establishing the synchronous LLC lung and flank tumor mouse model is shown in Fig. 5c. On day 7 post-RT, flow cytometry analysis of the distal lung TME revealed that the addition of Au/HA NPs to ablative RT significantly enhanced the infiltration of NK and cytotoxic T cells, with a trend toward an increased frequency of dendritic cells (Fig. 5d, e). Overall, the addition of Au/HA NPs to ablative RT effectively initiated both innate and adaptive immune responses and enhanced antitumor immunotherapeutic activity within the distant, non-irradiated lung TME, potentially contributing to an abscopal effect.

### 3.8 Biodistribution and toxicity profile of Au/HA NPs combined with ablative RT

**Fig. 6:**
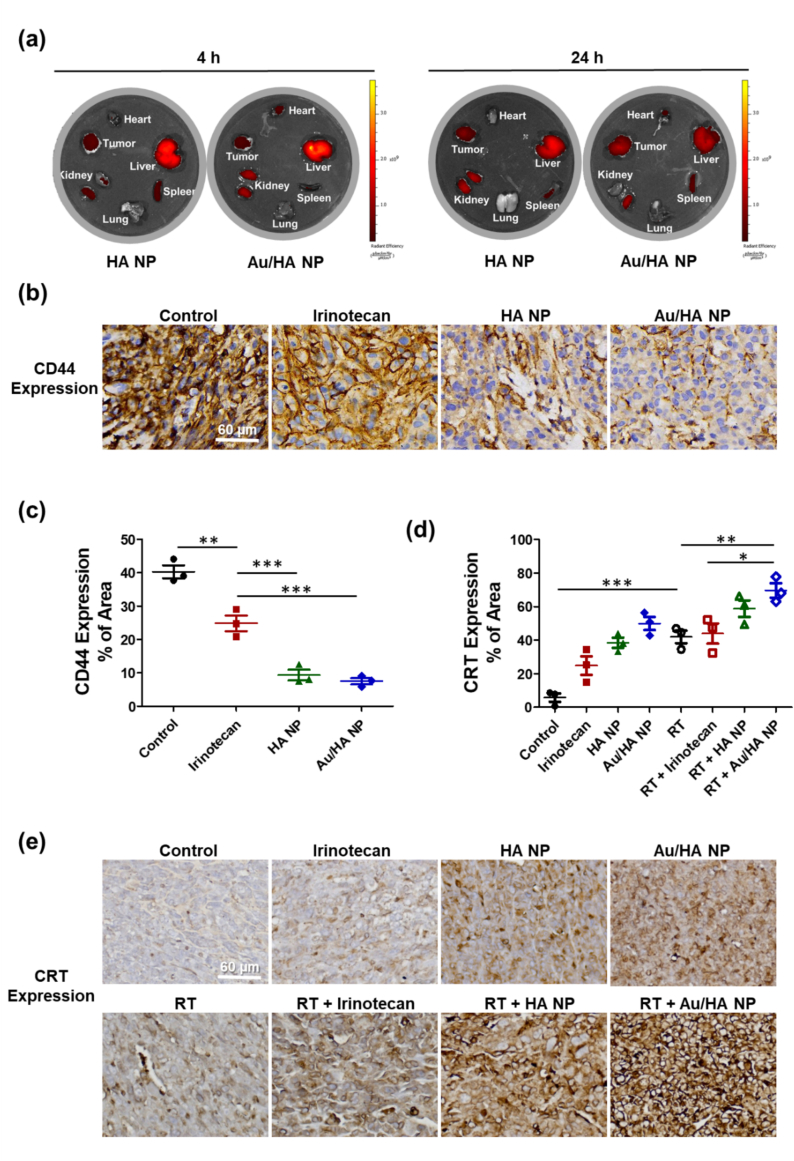
*In vivo* biodistribution of Au-HA-functionalized SN38 nanoparticles and CD44 and CRT expression in flank tumors. (a) Distribution of HA NPs and Au/HA NPs in the main organs and LLC tumors at 4 and 24 h. (b) Representative immunohistochemical images of CD44 expression in A549 (human adenocarcinoma) flank tumor tissue treated with HA NPs or Au/HA NPs. Scale bar = 60 μm. (c) Quantitative results for CD44 expression. The data represent the percentage of CD44-stained area within the observed area. Each experimental group involves three mice (n = 3). (d) Quantitative results for CRT expression. The data represent the percentage of CRT-stained areas within the observed area. Each experimental group involves three mice (n = 3). (e) Representative immunohistochemical images of CRT expression in LLC flank tumor tissue treated with radiotherapy alone or in combination with SN38, HA NPs, or Au/HA NPs. Scale bar = 60 μm. Data are expressed as the mean ± standard error of the mean. Statistical analyses are performed using the one-way analysis of variance with Tukey's multiple comparisons test. *P < 0.05; **P < 0.01; ***P < 0.001. Abbreviations: CRT, calreticulin; HA, hyaluronic acid; LLC, Lewis lung carcinoma; NP, nanoparticle; RT, radiotherapy.

To determine Au/HA NP biodistribution in LLC tumor-bearing mice, we intravenously injected Cy7.0-labeled HA NPs and Au/HA NPs, then excised and imaged the main organs and tumor tissues using an IVIS Imaging System. At 4 h post-injection, liver tissues in both the HA NP and Au/HA NP groups showed strong fluorescence intensity (Fig. 6a). This is consistent with the liver's role as a biological filtration system that captures 30–99 % of administered NPs from the bloodstream (Zhang et al., 2016). By 24 h post-injection, LLC tumor tissues exhibited increased Cy7.0 fluorescence (Fig. 6a), indicating effective NP accumulation within the tumor and supporting their controlled-release capability.

Mice receiving ablative RT alone or in combination with irinotecan, HA NPs, or Au/HA NPs exhibited normal serum levels of creatinine, blood urea nitrogen, alanine aminotransferase, and aspartate aminotransferase (Fig. S6a), supporting the absence of nephrotoxicity and hepatotoxicity. Additionally, no significant changes were observed in peripheral blood hematological parameters—including white blood cell counts, hemoglobin concentration, and platelet counts—demonstrating that the treatments did not induce hematological toxicity (Fig. S6b). Collectively, HA NPs or Au/HA NPs in combination with ablative RT were observed to be safe without significant hematological, hepatotoxic, or nephrotoxic effects. Nevertheless, individual monitoring of hematological and biochemical parameters is warranted.

### 3.9 ICD evaluation of Au/HA NPs combined with ablative RT

To determine whether the tumor growth inhibition observed with HA-targeted delivery was mechanistically linked to CD44 suppression, tumor specimens were subjected to immunohistochemical staining (Fig. 6b). In line with the results of the *in vivo* flow cytometric analysis, immunohistochemistry findings demonstrated that CD44 expression was significantly lower in the HA NP group than in the other treatment groups, confirming effective target engagement (Fig. 6c). CRT exposure is a critical early event that enhances tumor antigen presentation and potentiates antitumor immunity. Further, CRT expression was the highest in the combined ablative RT and Au/HA NP group (Fig. 6d, e). This supports that this regimen effectively triggers ICD and amplifies systemic immune responses.

## 4 Discussion

Our study demonstrated that HA-functionalized NPs, particularly Au/HA NPs, significantly enhanced the therapeutic efficacy of ablative RT in lung cancer through multiple synergistic mechanisms. HA conjugation facilitated receptor-mediated endocytosis, resulting in enhanced tumor cell internalization and improved cytotoxic delivery of SN38 (Kesharwani et al., 2021). Additionally, Au/HA NPs potentiated the radiosensitizing effect of ablative RT by amplifying radiation-induced DNA double-strand breaks, as demonstrated by elevated γH2AX expression, and promoting G2/M cell cycle arrest (Janic et al., 2021). This was accompanied by increased ROS production, CRT expression, and extracellular ATP release, leading to enhanced ICD and the initiation of systemic antitumor immune responses (Zhu et al., 2023). Immune profiling further revealed that combining ablative RT with Au/HA NPs significantly boosted both innate and adaptive immunity responses within the TME. The increased recruitment of NK, cytotoxic T, and dendritic cells suggested that the ICD triggered by this combination therapy orchestrated a potent antitumor immune response, effectively enhancing innate and adaptive immunities. Supporting the involvement of the immune system, quantitative analyses revealed a reduction in regulatory T cells and an increased CD8^+^/Treg ratio (Noyes et al., 2022). Notably, these immune-stimulatory effects extended beyond the irradiated site, with increased infiltration of effector immune cells in distal, non-irradiated lung tumors, supporting the potential for abscopal effects (Liu et al., 2021; Viswanath et al., 2024).

With respect to NP formulation, PEGylated Au NPs have been shown to evade reticuloendothelial system recognition, exhibit prolonged blood circulation, and enhance tumor accumulation (Wang et al., 2020). Similarly, bovine serum albumin (BSA)-coated Au NPs effectively minimize NP aggregation and extend systemic circulation. However, they have a risk of immunogenicity and potential inflammatory toxicity in normal tissues due to their animal protein origin (Jakic et al., 2024). Both PEG- and BSA-modified systems primarily rely on passive tumor accumulation through the enhanced permeability and retention effect (Leopold et al., 2017). In our study, we incorporated human serum albumin (HSA) as an NP component to maintain colloidal stability, reduce aggregation, and prolong circulation time while minimizing immunogenicity and normal tissue toxicity. Furthermore, HA functionalization provided active tumor targeting *via* specific receptor binding, in addition to favorable biocompatibility. Collectively, these design advantages positioned Au/HA NPs as a promising platform for tumor-selective radiosensitization.

Recent studies have investigated various metal–polymer hybrid NPs for radiosensitization and drug delivery in lung cancer. Chen et al. demonstrated that Alb-modified Au NPs exhibited potent radiosensitizing effects and significantly enhanced RT efficacy in lung cancer mouse model with minimal normal tissue toxicity (Chen et al., 2023a). However, they did not evaluate immunogenic cell death or TME immune amplification. Muradova et al. developed RGD-modified AGuIX-Bi NPs that actively targeted integrin-expressing lung cancer cells, enhancing NP uptake, radiosensitization, and ICD-associated immune activation. Ultimately, the tumor microenvironment was transformed into an immunostimulatory and T cell–inflamed state (Muradova et al., 2025). Our study integrates these therapeutic principles by employing HA-functionalized Au NPs that achieve receptor-mediated active targeting and chemotherapeutic delivery, further amplifying ICD induction and strengthening antitumor immunity.

Regarding the Au content of the designed nanoparticles, it is worth noting that the Au signals in the XPS spectra (Au 4f at 84 eV and Au 4d₅ at 335 eV) and XRD patterns appeared relatively weak. This observation is consistent with the specific composition of our nanoparticle formulation. Given the total mass of the components (0.5 mg SN38, 10 mg HSA, 0.078 mg HA, 0.078 mg PEI, and 0.0046 mg Au), the total nanoparticle mass is 10.6606 mg, yielding a theoretical gold content of approximately 0.043 % (*w*/w). While this low concentration approaches the detection limits of standard surface analysis techniques, it proved sufficient to drive significant biological outcomes. Our results demonstrate that even at this trace loading level, the Au/HA NPs effectively catalyzed ROS generation and enhanced radiosensitivity, leading to substantial tumor cell death.

The improved chemotherapeutic delivery observed with our HA-functionalized NPs was supported by increased cellular internalization and enhanced cytotoxicity *in vitro* and prolonged detectable tumor accumulation *in vivo*. The combination with RT led to improved tumor control. Although our data confirm CD44 receptor–mediated endocytosis as one mechanism of uptake (Kesharwani et al., 2021; Kim and Kumar, 2014), we acknowledge that additional processes may have also contributed. Particularly, the incorporation of HSA may have enhanced passive tumor accumulation through the enhanced permeability and retention (EPR) effect (Xiong et al., 2025). This provided a complementary route for NP delivery and controlled drug release. Collectively, the receptor-mediated internalization and HSA-associated EPR effects may underlie the improved tumor targeting and therapeutic responses observed.

NK cell subsets possess the unique capacity to eliminate tumor cells without the need for prior antigen sensitization, making them promising candidates for anticancer immunotherapy. Both NK and NKT cells are key players in enhancing the therapeutic effectiveness of ablative RT within the inflammatory TME (Patin et al., 2022). Our findings demonstrate that the combination of HA-functionalized NPs and ablative RT significantly promotes NK cell infiltration within the irradiated flank TME, indicating an amplified antitumor immune response. In addition to promoting the release of tumor-associated antigens and DAMPs, nanomedicine-assisted RT can also alleviate tumor hypoxia and enhance proinflammatory signaling, both of which help activate antitumor immune responses (Ó Murchú et al., 2025). Given the complexity and heterogeneity of immune responses elicited by Au NPs combined with RT, further comprehensive investigations are warranted to elucidate their immunomodulatory mechanisms.

In conclusion, our findings highlight the potential of CD44-targeted HA NPs, particularly Au/HA NPs, as an effective strategy to enhance the therapeutic benefits of ablative RT. By simultaneously enhancing radiosensitization, targeted drug delivery, and ICD with immune activation, this approach promises improved lung cancer treatment outcomes. Further studies should focus on refining NP formulations, elucidating underlying immunomodulatory mechanisms, and validating efficacy in clinically relevant models to advance this strategy toward clinical application.

## Declaration of generative AI and AI-assisted technologies

The authors did not use generative AI and AI-assisted technologies in the manuscript preparation process.

## CRediT authorship contribution statement

**Jenny Ling-Yu Chen:** Writing – original draft, Software, Resources, Project administration, Methodology, Investigation, Funding acquisition, Formal analysis, Data curation, Conceptualization. **Shu-Jyuan Yang:** Writing – original draft, Software, Resources, Methodology, Investigation, Formal analysis, Data curation, Conceptualization. **Li-Cheng Lin:** Software, Resources, Project administration, Methodology, Investigation, Formal analysis, Data curation. **Chun-Kai Pan:** Resources, Project administration, Methodology, Investigation. **Ching-Yi Tsai:** Software, Resources, Project administration, Methodology, Investigation. **Yu-Sen Huang:** Writing – review & editing, Visualization, Validation, Supervision, Software, Resources, Project administration, Funding acquisition. **Ke-Cheng Chen:** Visualization, Validation, Supervision, Software, Resources. **Ming-Jium Shieh:** Writing – review & editing, Visualization, Validation, Supervision, Software, Resources. **Yu-Li Lin:** Writing – review & editing, Visualization, Validation, Supervision, Software, Resources, Project administration, Methodology, Formal analysis, Conceptualization.

## Funding

This work was supported by the 10.13039/501100005762National Taiwan University Hospital [grant number NTUH-114-SS0007] and the Taiwan 10.13039/100007225Ministry of Science and Technology [grant number NSTC 113–2314-B-002-158 and grant number NSTC 114-2320-B-002-017-MY3].

## Declaration of competing interest

The authors report there are no competing interests to declare.

## Data Availability

The authors confirm that the data supporting the findings of this study are available within the article and its supplementary materials.

## Appendix A Supplementary data

Supplementary material Image 1
