# Supplementary material for: Gold-incorporated hyaluronic acid nanoparticles enhance ablative radiotherapy efficacy in lung cancer
Source: Int J Pharm X. 2025 Dec 23;11:100480. doi: 10.1016/j.ijpx.2025.100480 (PMC12808585; doi:10.1016/j.ijpx.2025.100480)
Supplement: Supplementary file 1 — Supplementary material [file mmc1.docx]

**Supplementary Materials**

**Gold-incorporated Hyaluronic Acid Nanoparticles Enhance Ablative Radiotherapy Efficacy in Lung Cancer**

Jenny Ling-Yu Chen, Shu-Jyuan Yang, Li-Cheng Lin, Chun-Kai Pan, Ching-Yi Tsai, Yu-Sen Huang, Ke-Cheng Chen, Ming-Jium Shieh, Yu-Li Lin

**Table of Contents**

| **Supplementary Table S1** |  |
| --- | --- |
| Compositions of the HA-functionalized SN38 NPs and Au/HA NPs | 2 |
| **Supplementary Table S2** |  |
| IC_50_ values with 95% CIs for SN38, HA-functionalized SN38 NPs, and Au/HA NPs | 3 |
| **Supplementary Figure S1** |  |
| Size distributions of NPs across batches | 4 |
| **Supplementary Figure S2** |  |
| Storage and colloidal stabilities of NPs as assessed according to the polydispersity index | 5 |
| **Supplementary Figure S3** |  |
| Cellular internalization of HA-functionalized SN38 NPs and Au/HA NPs | 6 |
| **Supplementary Figure S4** |  |
| Intracellular ROS generation | 7 |
| **Supplementary Figure S5** |  |
| Quantitative flow cytometric analysis of subpopulations of tumor-infiltrating immune cells in the flank tumor microenvironment | 8 |
| **Supplementary Figure S6** |  |
| Biochemical and hematological parameters in the peripheral blood of mice recorded 7 days after ablative RT | 9 |

**Supplementary Table S1.** Compositions of the HA-functionalized SN38 NPs and Au/HA NPs

| Group | HSA | SN38 | PEI | HA | Au NPs |
| --- | --- | --- | --- | --- | --- |
| HA NPs | ✓ | ✓ | ✓ | ✓ |  |
| Au/HA NPs | ✓ | ✓ | ✓ | ✓ | ✓ |

Abbreviations: HA, hyaluronic acid; HSA, human serum albumin; NPs, nanoparticles; PEI, polyethyleneimine

**Supplementary Table S2.** IC_50_ values with 95% CIs for SN38, HA-functionalized SN38 NPs, and Au/HA NPs

|  |  | SN38 | HA NP | Au/HA NP |
| --- | --- | --- | --- | --- |
| A549 | IC_50_ (ng/mL) | 119.7 ± 10.8 | 106.1 ± 10.0 | 108.4 ± 2.8 |
|  | 95% CI (ng/mL) | 103.5 to 139.4 | 85.2 to 131.4 | 91.6 to 129.3 |
| H226 | IC_50_ (ng/mL) | 20.4 ± 2.3 | 16.6 ± 1.6 | 15.4 ± 1.7 |
|  | 95% CI (ng/mL) | 11.7 to 28.2 | 11.9 to 21.0 | 10.5 to 19.3 |
| LLC | IC_50_ (ng/mL) | 12.8 ± 0.7 | 10.4 ± 0.1 | 10.4 ± 0.2 |
|  | 95% CI (ng/mL) | 11.7 to 13.9 | 9.4 to 11.4 | 10.0 to 10.9 |

Data are shown as the mean ± standard deviation of three independent experiments.

Abbreviations: CI, confidence interval; HA, hyaluronic acid; NPs, nanoparticles

**
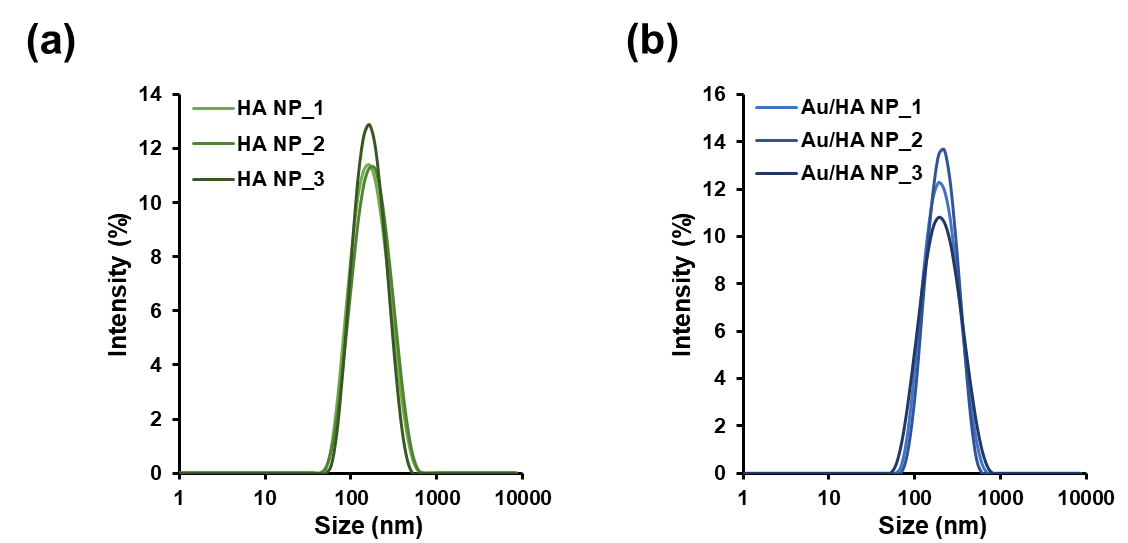
**

**Fig. S1.** Size distributions of NPs across batches. **(a)** Size distributions of HA-functionalized SN38 NPs across three independent batches. **(b)** Size distributions of Au/HA NPs across three independent batches.

Abbreviations: HA, hyaluronic acid; NPs, nanoparticles

**
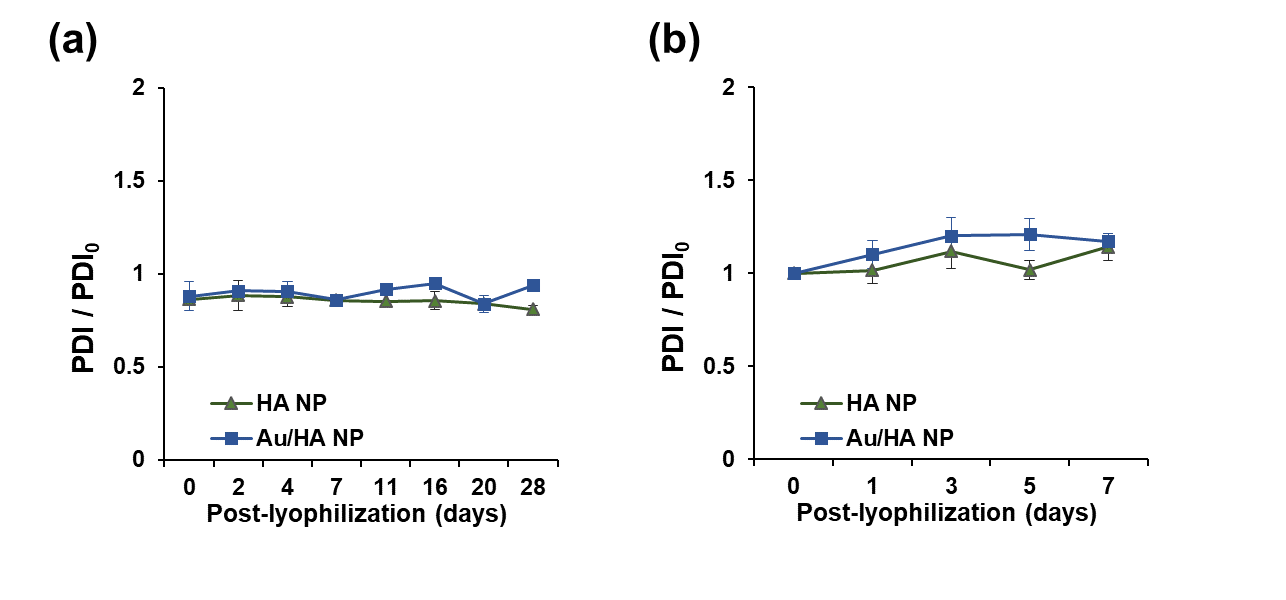
**

**Fig. S2.** Storage and colloidal stabilities of NPs as assessed according to the polydispersity index. **(a)** Storage stability of NPs as measured according to the PDI is determined by dissolving the re-lyophilized HA-functionalized SN38 NPs and Au/HA NPs in 1 mL double-distilled water (ddH_2_O). **(b)** Colloidal stability of NPs as measured according to PDI is assessed in Dulbecco’s Modified Eagle Medium (Gibco) supplemented with 10% (v/v) fetal bovine serum to evaluate stability under physiologically relevant conditions. Each experimental group includes five samples (n = 5). Data are expressed as the mean ± standard error of the mean.

Abbreviations: HA, hyaluronic acid; NP, nanoparticle; PDI, polydispersity index


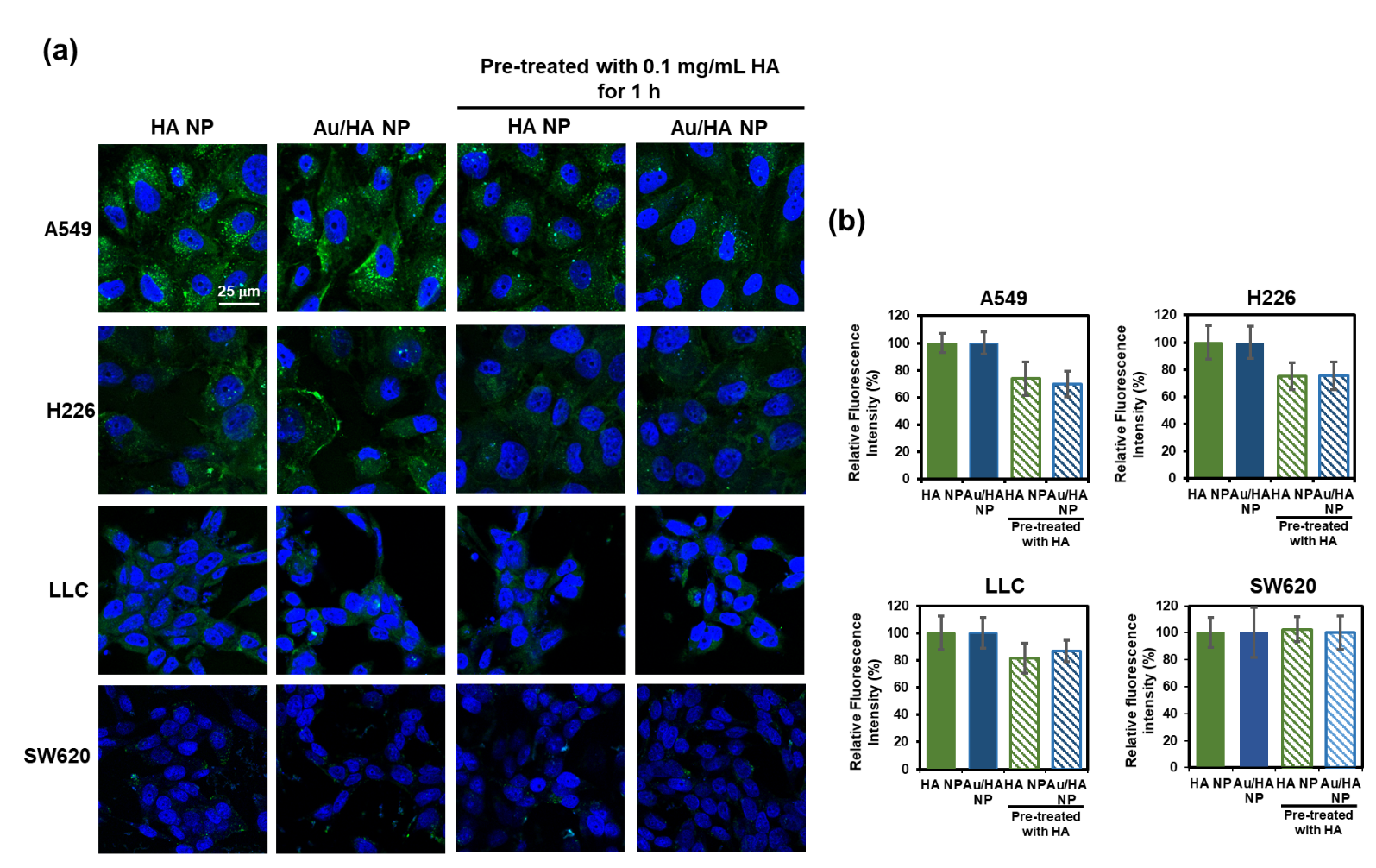


**Fig. S3.** Cellular internalization of HA-functionalized SN38 NPs and Au/HA NPs. **(a)** Representative fluorescence merged images of FITC-labeled HA NPs and Au/HA NPs (shown in green) and nuclei (shown in blue) (Hoechst 33342) in A549, H226, Lewis lung carcinoma (LLC), and SW60 cells with or without 0.1 mg/mL HA pretreatment. **(b)** Quantification of green fluorescence intensity in A549, H226, LLC, and SW60 cells treated with HA NPs or Au/HA NPs with or without 0.1 mg/mL HA pretreatment. Green fluorescence is quantified from six randomly selected regions per sample (n = 6). Data are expressed as the mean ± standard error of the mean.

Abbreviations: HA, hyaluronic acid; NP, nanoparticle

**
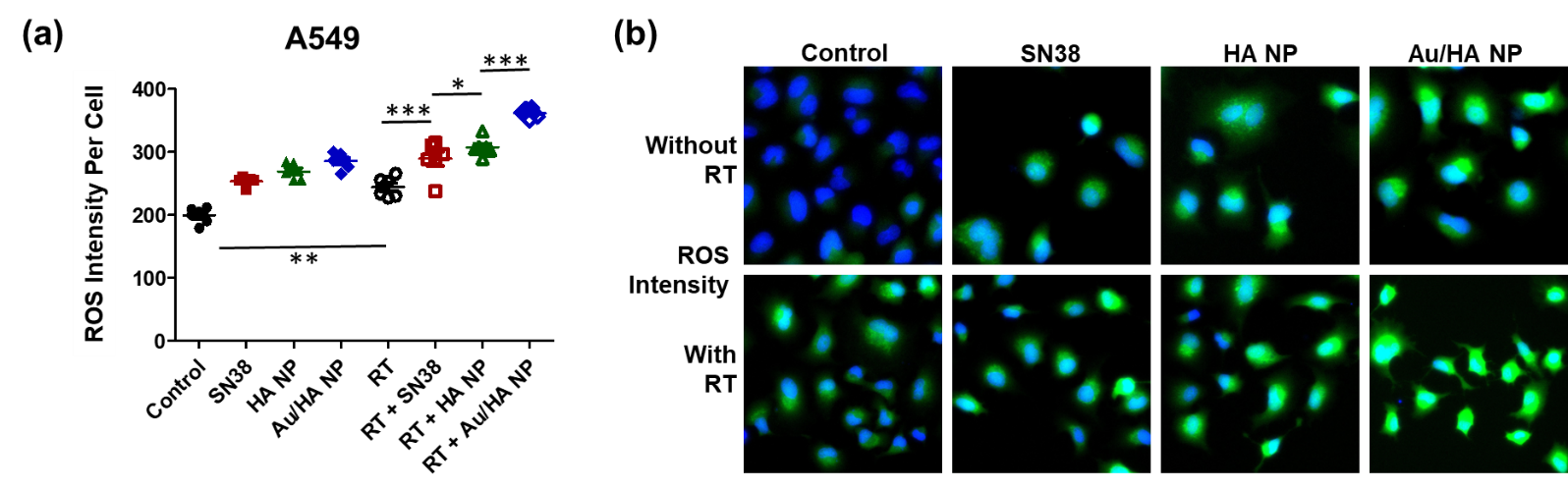
**

**Fig. S4.** Intracellular ROS generation. ROS generation induces nuclear damage and triggers immunogenic cell death. Intracellular ROS are detected by DCFH-DA staining. **(a)** Quantification of ROS fluorescence intensity per cell in A549 cells treated with SN38, HAfunctionalized SN38 NPs, Au-HA NPs, or RT (6 Gy). Each experimental group includes six samples (n = 6). **(b)** Representative fluorescence merged images of ROS (shown in green) and nuclei (shown in blue) (Hoechst 33342) in A549 cells treated with SN38, HA NPs, or Au/HA NPs and RT (6 Gy). Data are expressed as the mean ± standard error of the mean. Statistical significance is assessed using one-way analysis of variance with Tukey’s multiple comparisons test. **P*<0.05; ***P*<0.01; ****P*<0.001.

Abbreviations: HA, hyaluronic acid; NP, nanoparticle; ROS, reactive oxygen species; RT, radiotherapy


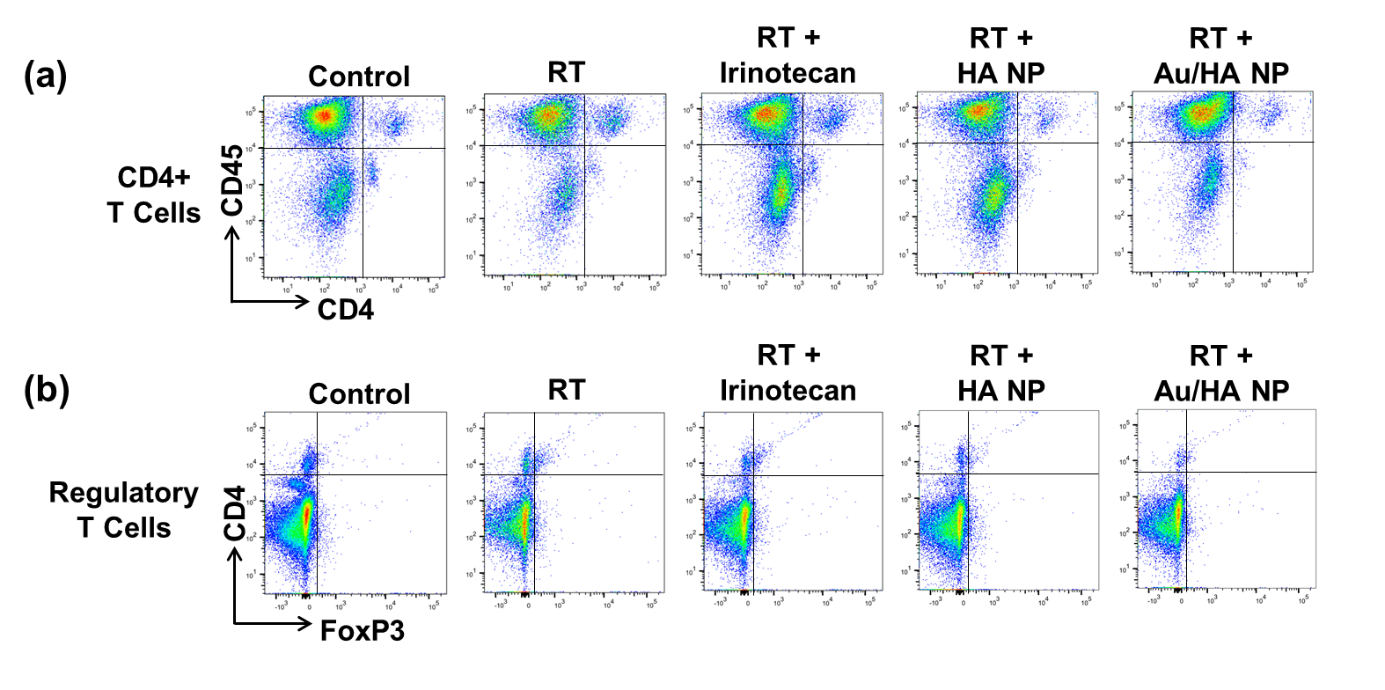


**Fig. S5.** Quantitative flow cytometric analysis of subpopulations of tumor-infiltrating immune cells in the flank tumor microenvironment. C57BL/6 mice undergo subcutaneous implantation of murine LLC cells in the ﬂanks and are randomized to one of the following treatment groups: irinotecan (20 mg/kg on day 0, administered intravenously), hyaluronic acid SN38 nanoparticles (HA NPs, 20 mg SN38/kg on day 0, administered intravenously), or Au-incorporated HA SN38 NPs (Au/HA NPs, 20 mg SN38/kg on day 0, administered intravenously), with or without ablative RT (12 Gy/day for 2 days on days 0 and 1) to the ﬂank tumor. Tumor-inﬁltrating immune cells are isolated from the ﬂank tumor microenvironment to obtain cell suspensions for surface staining. Representative flow cytometric analysis of **(a)** CD4^+^ T cells and **(b)** CD4^+^ FoxP3^+^ regulatory T cells is shown.

Abbreviations: HA, hyaluronic acid; LLC, Lewis lung carcinoma; NP, nanoparticle; RT, radiotherapy

**
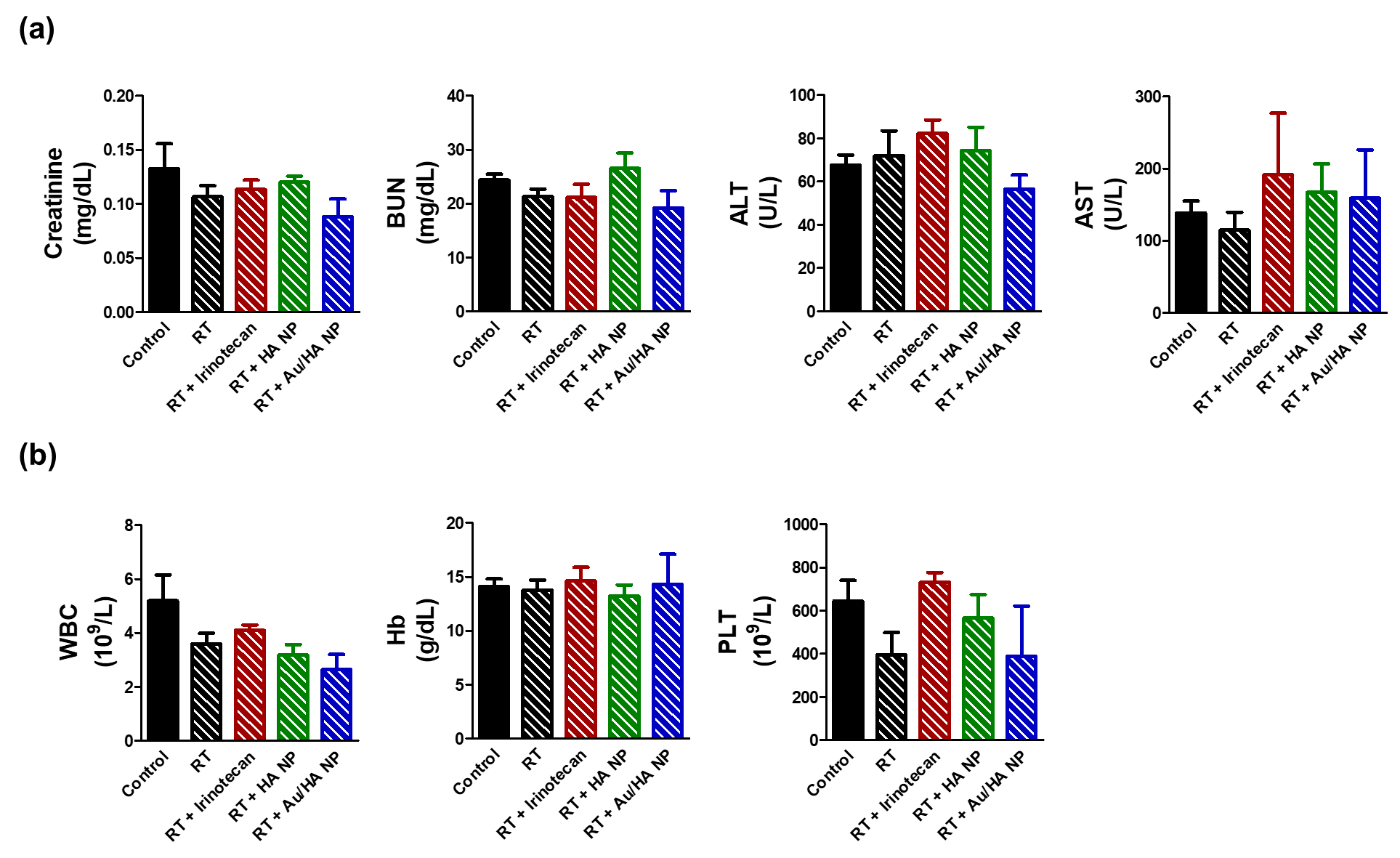
**

**Fig. S6.** Biochemical and hematological parameters in the peripheral blood of mice recorded 7 days after ablative RT. (**a**) Creatinine, BUN, ALT, and AST. (**b**) WBC, Hb, and PLT count. Data are expressed as the mean ± standard error of the mean. Each experimental group includes five mice per group (n = 5).

Abbreviations: ALT, alanine aminotransferase; AST, aspartate aminotransferase; BUN, blood urea nitrogen; HA, hyaluronic acid; Hb, hemoglobin; NP, nanoparticle; PLT, platelet; RT, radiotherapy; WBC, white blood cell count
